# Method and Application for Dynamic Comprehensive Evaluation with Subjective and Objective Information

**DOI:** 10.1371/journal.pone.0083323

**Published:** 2013-12-26

**Authors:** Dinglin Liu, Xianglian Zhao

**Affiliations:** College of economic and management, Nanjing University of Aeronautics and Astronautics, Nanjing, Jiangsu, China; Universidad Veracruzana, Mexico

## Abstract

In an effort to deal with more complicated evaluation situations, scientists have focused their efforts on dynamic comprehensive evaluation research. How to make full use of the subjective and objective information has become one of the noteworthy content. In this paper, a dynamic comprehensive evaluation method with subjective and objective information is proposed. We use the combination weighting method to determine the index weight. Analysis hierarchy process method is applied to dispose the subjective information, and criteria importance through intercriteria correlation method is used to handle the objective information. And for the time weight determination, we consider both time distance and information size to embody the principle of esteeming the present over the past. And then the linear weighted average model is constructed to make the evaluation process more practicable. Finally, an example is presented to illustrate the effectiveness of this method. Overall, the results suggest that the proposed method is reasonable and effective.

## Introduction

Over the years, dynamic comprehensive evaluation has been of great importance in the comprehensive evaluation theory. Compared with the static comprehensive evaluation, dynamic comprehensive evaluation, which studies on the performances of evaluated objects in a certain time period, faces with more complicated situations [1]. Due to its advantages, dynamic comprehensive evaluation has become increasingly attractive for applications such as economic and management [2]. Furthermore, dynamic comprehensive evaluation is a complex process in which a variety of information needs to be processed. So how to make full use of the information during the dynamic evaluation process has become the worth studying area at present.

In the specialized literature there is a considerable amount of research on the methods or applications of the dynamic comprehensive evaluation. The early studies focused on dealing with the real closure of an order field [3], [4]. And then the evaluation process has improved through different ways, such as considering value fluctuation [5], object gain [6] or background [7], scattering degree [8], [9], as well as programming [10]. At the same time, how to calculate the index or time weight more accurately has become central to optimize the dynamic comprehensive evaluation [11]–[13]. Moreover, the trend of methods developing toward multiplicity has also promoted the application research of dynamic comprehensive evaluation [14]–[16]. However, as clearly studied on index weight, many researchers have mainly ascertained it by applying subjective or objective information, but few researchers have used both [17]. For the time weight many studies have been published concerning on the time distance, while little attention has been paid to the weight changes caused by the index information [18], [19].

This study therefore aims to present a dynamic comprehensive evaluation method to make heave use of subjective and objective information. In particular, (1) a combination weighting method is adopted to determine the index weight, including analysis hierarchy process (AHP) method to deal with subjective preference information, and criteria importance through intercriteria correlation (CRITIC) method to dispose the objective data; (2) both time distance and information size are applied to embody the different importance of each time point; (3) furthermore, a linear weighted average model is constructed by stating the optimal ordinal method; (4) and finally, comparing the results with the previous ones in ref. [15], the effectiveness of this method has been illustrated clearly.

The organization of the rest of the paper is as follows. In section 2 the research problem is briefly described. The index and time weights confirmation are presented in section 3. In section 4 we briefly describe the linear weighted average model for dynamic comprehensive evaluation. We illustrate the effectiveness of the method in this study through an example in section 5. The final section concludes the research results and puts forward the future work.

## Problem description

Let 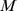 be a set which contains 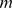 indicators including 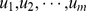. The set 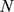 is a set which has 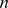 evaluation objects 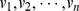. The index weight set is 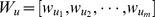, and 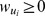 with 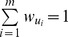. The set 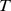 is on behalf of the evaluation period from 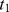, 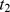 to 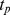, and the time weight set is 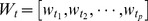 with 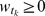 and 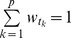. Under the index 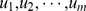, the evaluation research about objects 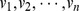 during time period 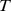 forms a dynamic comprehensive evaluation problem.


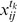
 stands for the observed value of object 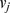 (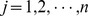) under indicator 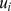 (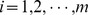) at time 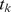 (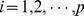). The data unification and dimensionless should be stated in the first place [20]–[23], because there may be distinct in type, unit or order of magnitudes among the indicators. After the data preprocessing, we assume that 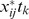 presents the standard data used in this study.

## Weight determination

### Index weight determination

#### Part 1, the initial weight determination: with AHP method

AHP method developed by T. L. Satty et al. [24]–[26], constructs a hierarchical structure model to determine the importance degree of index. In this study, evaluators apply 1–9 scale method to compare the importance of each indicator [27]. And the data reflects the subjective preference information of evaluators. By using the subjective preference information, the analytical procedures are as follows:

1. The analytic hierarchy structure is constructed. The index set 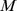 is decomposed into different subsets including certain indicators. And its subordinate relations are confirmed according to the relationship between indicators.
2. The evaluation index comparison judgment matrix 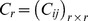 is confirmed. 
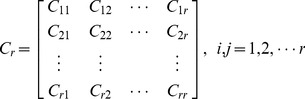
(1)where 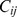 is the important value after comparing 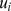 with 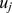, and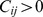, 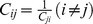 with 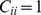.
3. Single hierarchy sorting and consistency check are affirmed. The characteristic roots of judgment matrix 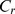 are calculated. After normalizing the characteristic roots, the weights of each indictor in the same evaluation level can be concluded. And then the random consistency ratio 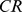 is confirmed. 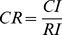
(2)

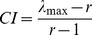
, 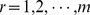. 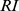
is the average random consistency index. If 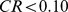, the sorting has satisfactory consistency.
4. The initial weight, which reflects the subjective information, is determined. The weight of every indicator is calculated at all levels; then the initial weight 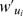 of indicator 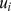 is determined. And 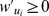, with 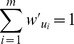.

#### Part 2, the secondary weight determination: with CRITIC method

CRITIC method, which proposed by Diakoulaki in 1995 [28], reflects the relative importance by applying the comparative and conflict information among the indicators. So CRITIC method is chosen to dispose the objective information that refers to the observed value of evaluation object under the index. And its procedures are as follows:

1. The conflicts between 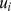 and 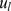 at time 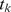 are quantized. 
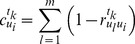
(3)where 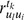 is the correlation coefficient between 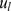 and 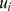 at 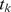.
2. The information quantity of 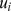 at time 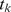 is confirmed. 
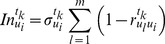
(4)where 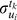 is the standard deviation of 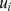 at 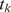.
3. The weight of 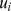 at time 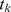 is calculated. 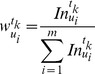
(5)
4. The secondary weight is determined. The average index weight of 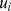 in time phase 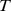 is 
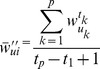
(6)where 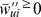, and 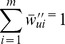.

#### Part 3, the index weight determination: with combination weighting method

In this process, we combine the weights, which are confirmed by both AHP and CRITIC methods, to determine the index weight.

Definition 1. The index weight which contains both subjective and objective information is 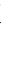
(7)where 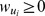, and 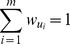.

The index weight 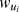 on the one hand contains the subjective preference of evaluators, and on the other hand reflects the objective information of each indicator. If 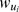 is close to 0, indicator 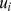 is less significant, while if 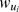 is close to 1, the importance of 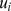 is larger. Above all, the index weight 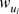 realizes effectively about the combination of both subjective and objective information.

### Time weight determination

We deem that the time weight 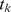 (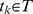) significantly associates with two factors: time distance and information size. The former one means that new information has greater importance than the old one, and the latter one implies that the lager the information size of each indicator at time 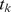, the more important 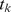 is.

Definition 2. We define that the weight of time 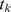 is 
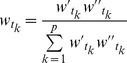
(8)where 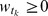, and 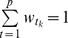.

In formula (8) 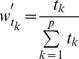
(9)

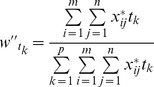
(10)where 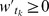, and 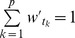, with 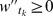, and 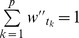.

The time weight reflects both the time distance and index information. If 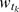 is close to 0, time 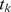 becomes less crucial; while if 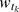 is close to 1, the importance of 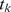 is greater. In a word the time weight embodies the principle of esteeming the present over the past and reflects the importance of information.

## Model

### The linear weighted average model

To make the evaluation process more practicable, we construct the dynamic comprehensive evaluation model by introducing the optimal ordinal method.

For 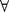 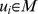, we set 
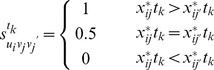
(11)where 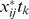 is the standardized data of object 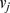 under indicator 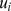 at time 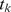, and 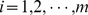, 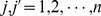, with 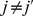 [29].

Definition 3. The optimal ordinal of 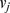 at time 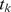 by comparing with the other 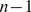 evaluation objects is 
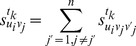
(12)


Definition 4. The total optimal ordinal of 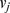 at time 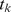 by comparing with other 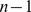 evaluation objects is 
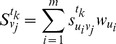
(13)


The linear weighted average model is 
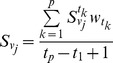
(14)


From the formula (14), we know that 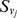 is the comprehensive evaluation value which is determined by the linear weighted average model. Whether 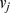 plays well or not in the time phase 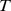 is known clearly by comparing 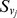 with other objects.

## Example analysis

We evaluate the regional environmental risk in China from 2003 to 2007 by using the same evaluation index, information, data pretreatment method and so on in ref. [15]. And furthermore, we compare the evaluation results with the ones in ref. [15] to illustrate the effectiveness of the above method. The original data is collected in China Statistical Yearbook (2004–2008) and China Environmental Yearbook (2004–2008). The specific calculation processes are as follows:

1. The same extremum method in ref. [15] is adopted to standardize the initial data;
2. The index weight is determined. For the subjective information, we apply the same one which was calculated by AHP method in ref. [15]. The objective information is computed by formulae (3)–(6) of CRITIC method. The combination of subjective and objective weights is determined finally by using the formula (7).
3. The time weight of each evaluation year is confirmed by utilizing formulae (8)–(10). 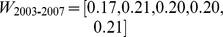.
4. The comprehensive evaluation values are calculated. We apply the formulae (11)–(14) to calculate the comprehensive evaluation value, and finally sort the evaluation objects.
5. Compared the evaluation results with the ones in ref. [15].

Table 1 shows that there are many differences between subjective and objective weights. Subjective weight has on behalf of the evaluators, and objective weight reflects the data information of index. From table 1, it is observed that the combination of subject and object weights effectively states the significance of index.

**Table 1:** The results of index weight.

Index	Subjective weight	Objective weight	Combination weight
Number of pollution and destruction accidents	0.12	0.28	0.21
SO_2_ per unit area	0.18	0.10	0.11
The ratio of COD emissions and environmental capacity	0.30	0.22	0.41
Population density	0.20	0.10	0.12
Economic density	0.14	0.10	0.08
The ratio of nature reserve	0.06	0.21	0.08

Table 2 displays the dynamic comprehensive evaluation results in this study. Basing on the evaluation values, it is concluded the rank ordering of environmental risk of each provinces; then 31 provinces in China are divided into four risk types. Shanghai, Tianjin, Shandong, Beijing, Jiangsu and Liaoning fall into the category of type IV which has the high environment risk. The evaluation values of 10 provinces between 3.00 and 4.00, and they have a less high risk. And 11 provinces including Hunan, Gansu, Chungking and so forth are a form of type II which means a less low risk. Yunnan, Sinkiang, Chinghai and Tibet, whose evaluation values are much lower than others', are classified as type I which refers to the low risk.

**Table 2:** The dynamic comprehensive evaluation results.

Provinces	Evaluation values	No.	Risk type
Beijing	4.52	4	IV
Tianjin	4.69	2	IV
Hebei	3.98	7	III
Shanxi	3.71	9	III
Inner Mongolia	2.33	22	II
Liaoning	4.15	6	IV
Jilin	2.69	20	II
Heilongjiang	2.50	21	II
Shanghai	5.10	1	IV
Jiangsu	4.43	5	IV
Zhejiang	3.62	10	III
Anhui	3.12	14	III
Fujian	2.12	27	II
Jiangxi	2.30	23	II
Shandong	4.54	3	IV
Henan	3.82	8	III
Hubei	3.14	13	III
Hunan	2.94	17	II
Guangdong	3.46	11	III
Guangxi	3.00	16	III
Hainan	2.07	28	II
Chungking	2.84	19	II
Sichuan	2.14	24	II
Guizhou	2.12	25	II
Yunnan	1.82	28	I
Tibet	0.61	31	I
Shaanxi	3.11	15	III
Gansu	2.93	18	II
Chinghai	0.71	30	I
Ningxia	3.30	12	III
Sinkiang	1.17	29	I

Type IV refers to high risk, type III refers to a less high risk, type II refers to a less low risk, and type I refers to low risk.

Table 3 exhibits the dynamic comprehensive evaluation results in ref. [15]. Contracted with the evaluation results in ref. [15], the environment risk classification of 31 provinces in China has different degrees of variation in our study. Further analysis of the results, we suggest that the division of environment risk is much more detailed than the one in ref. [15]. Beijing had the high risk and in the third place in ref. [15], while the order is higher than the one in this research. In our study, the rank of Beijing is much more coincided with the actual environment improvement condition from 2003 to 2007. In addition, Jiangsu, Shandong and Liaoning developed rapidly and the environment destructions had increased and so they should be in a high risk at that period. For Yunnan, Sinkiang, Chinghai and Tibet, the damages of the environment were low and they were in the low risk level. While for the other 12 provinces in low risk type in ref. [15], the environment had been destroyed much more than the remaining four provinces' - Yunnan, Sinkiang, Chinghai and Tibet. So it is not reasonable to put the remaining 12 provinces in the low risk type in ref. [15].

**Table 3:** The dynamic comprehensive evaluation results in ref. [15].

Risk type	Provinces
High risk	Tianjin, Shanghai, Beijing
Medium risk	Hebei, Jiangsu, Shandong, Ningxia, Zhejiang, Henan, Shanxi, Liaoning, Guangdong, Chungking, Guangxi, Hunan
Low risk	Anhui, Hubei, Guizhou, Shaanxi, Fujian, Jiangxi, Sichuan, Gansu, Yunnan, Jilin, Hainan, Heilongjiang, Inner Mongolia, Tibet, Chinghai, Sinkiang

The provinces in each risk type are ordered from large to small according to their evaluation values.

The data provide evidence that the results of this study are more in line with reality than the ones in ref. [15]. The reason why the evaluation results are more elaboration is because both subjective and objective information, which embody in the index and time weights, are applied. Consequently, we consider that the above method makes up for the information insufficient in ref. [15], and to some extent it is more reasonable and effective.

## Conclusions and future work

In this paper, we propose a dynamic comprehensive evaluation method with subjective and objective information. The combination weighting method (AHP and CRITIC methods), which applies much more data information, has improved the accuracy of index weight. The time weight has reflected the principle of esteeming the present over the past by considering both time distance and information size in each time point. We construct the dynamic comprehensive evaluation model by introducing the optimal ordinal method. And the advantage of corresponding to reality of the proposed method has known clearly after compared with the results in ref. [15]. In all, we argue that the dynamic comprehensive evaluation method with subjective and objective information may have an effective and reasonable evaluation results.

However, there are still some limitations in this research. Possible future research topics can be stating nonlinear programming method into the dynamic comprehensive evaluation process.
