# Correction: Method and Application for Dynamic Comprehensive Evaluation with Subjective and Objective Information

**DOI:** 10.1371/journal.pone.0090260

**Published:** 2014-02-18

**Authors:** The PLOS ONE Staff

In part 3 of the Weight Determination section, there was an error in equation 7 titled "Definition 1. The index weight which contains both subjective and objective information"

Please view the correct equation 7 here:
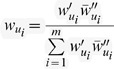
where 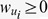, and 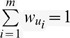.

## References

[1] Liu D, Zhao X (2013) Method and Application for Dynamic Comprehensive Evaluation with Subjective and Objective Information. PLOS ONE 8(12): e83323 doi:10.1371/journal.pone.0083323 24386176 10.1371/journal.pone.0083323 PMC3873306

